# Early Preferential Responses to Fear Stimuli in Human Right Dorsal Visual Stream - A Meg Study

**DOI:** 10.1038/srep24831

**Published:** 2016-04-20

**Authors:** Hanneke K. M. Meeren, Nouchine Hadjikhani, Seppo P. Ahlfors, Matti S. Hämäläinen, Beatrice de Gelder

**Affiliations:** 1 Cognitive and Affective Neuroscience Laboratory, Tilburg University, Tilburg, The Netherlands; 2 MGH/MIT/HMS Athinoula A. Martinos Center for Biomedical Imaging, Charlestown, MA, USA; 3 Gillberg Neuropsychiatry Center, Gothenburg University, Sweden; 4 Harvard-MIT Health Sciences and Technology, Cambridge, MA, USA; 5 Faculty of Psychology and Neuroscience, Maastricht University Maastricht Brain Imaging centre, M-BIC Oxfordlaan 55, 6229 ER Maastricht, The Netherlands

## Abstract

Emotional expressions of others are salient biological stimuli that automatically capture attention and prepare us for action. We investigated the early cortical dynamics of automatic visual discrimination of fearful body expressions by monitoring cortical activity using magnetoencephalography. We show that right parietal cortex distinguishes between fearful and neutral bodies as early as 80-ms after stimulus onset, providing the first evidence for a fast emotion-attention-action link through human dorsal visual stream.

When a person shows fear, bystanders spontaneously prepare to react to possible danger, indicating that whole body expressions of emotions automatically call for attention and trigger an adaptive response^1^. It is however not clear how our brain achieves the earliest differentiation of emotional content, which such rapid reactions seem to illustrate. In the well-established ventral and dorsal processing streams of the visual system, the temporal cortex engages in object recognition, including emotional stimuli, and the parietal cortex mediates computations for action^2^, as well as attention^3^. Many studies have shown that emotional stimuli elicit enhanced activation in temporal cortex, but recent functional magnetic resonance imaging (fMRI) research has also begun to highlight a potential role for the dorsal route^4^,^5^, thereby providing evidence for the close link between emotion and action envisaged by Darwin^1^. However, crucial information about the timing of neural events is necessary to substantiate the hypothesis that the emotion-action link runs through dorsal stream, but this information is currently still missing. Event related potential studies have suggested that the visual cortex is already sensitive for emotional body language around 100-ms after stimulus onset^6^, but its exact cortical origin remains unknown.

We investigated the cortical dynamics mediating early differentiation of fearful vs. neutral body expressions using magnetoencephalography (MEG) because it combines temporal resolution at the millisecond scale with good cortical spatial resolution. Event-related magnetic fields (ERF) were recorded using a 306-channel MEG system while healthy human volunteers watched greyscale photographs of human bodies expressing fear (fear condition) and performing a neutral action (neutral condition) (Methods).

The overall signal strength of the ERFs at the sensor level - mean global field power measured at the planar gradiometers - was significantly larger for upright fearful as compared to upright neutral bodies (one-tailed paired t-test, *P* = 0.01) around 100-ms after stimulus onset (Fig. 1), hereby confirming the early fear sensitivity found in event-related potential (ERP) studies^6^.

The cortical sources underlying these early differences were estimated on the cortical mantle of each individual subject^7^ (Methods). Statistical inferences were made by performing a non-parametric spatiotemporal cluster analysis^8^ on the entire cortex, hence taking care of the multiple comparison problem in both space and time.

A significant cluster (*P* = 0.012) was found for the upright fear > upright neutral contrast in the 80–110-ms time window in the right parietal cortex (Fig. 2). The spatial extent of this cluster included the cortical regions of the (anterior half of the) intraparietal sulcus (IPS), the postcentral sulcus (PoCS), and the inferior parietal lobule (IPL, including angular gyrus (AG) and supramarginal gyrus (SMG)). The parietal area identified by source localisation is consistent with reports from previous fMRI studies of perception of fearful body postures^4^,^5^,^9^,^10^.

Importantly, the early fear-effect was only found for images shown in upright orientation, and was absent when the same stimuli were inverted (see also Fig. 2C), indicating that the fear-effect is not caused by low-level visual properties, but rather by the emotional content that is no longer accessible when images are vertically inverted.

In summary, we found that the right lateral parietal cortex responded preferentially to fearful as compared to neutral whole body expressions between 80 and 110 ms after stimulus onset, whereas no such early differential activity could be found in the classical object recognition system of the occipitotemporal cortex.

This is the first report of such rapid parietal responses to complex natural stimuli. The observed cortical locations point to the activation of both the dorso-dorsal (Superior Parietal Lobule, e.g. IPS and PoCS) and the ventro-dorsal stream^11^ (IPL, e.g. AG and SMG) by the fearful content of the stimuli, thus providing rapid action understanding and action preparation^12^. Notably, the present response latency to fearful bodies is only half of that previously found in IPL for watching neutral body actions^13^.

In addition, the right IPL and AG are part of the ventral fronto-parietal attention network which (re) directs attention to salient stimuli^3^. The present response timing is consistent with the early time window during which transcranial magnetic stimulation of right parietal cortex disrupts spatial attention^14^ to simple stimuli. Interestingly, the pulvinar, a structure shown to selectively respond to life-threatening stimuli^15^, projects directly to the IPL^16^.

A recent rTMS study by Engelen *et al*.^17^ has shown a causal contribution of IPL in visual recognition of fearful body postures. This is a clear indication of the possible functional relevance of the present parietal modulation to visual perception. Recently two single- and paired-pulse TMS studies showed that different sectors of the motor system in the frontal lobe are specifically modulated during the sight of fearful as compared to neutral or happy body postures. Remarkably, these fear-specific modulations occurred in the same temporal window highlighted in the present study. Borgomaneri *et al*.^18^ showed a modulation of downstream cortico-spinal projections controlling the hand at 70–90 ms after visual stimulus onset. Furthermore, Borgomaneri *et al*.^19^ showed a modulation of intracortical facilitatory mechanisms in the motor cortex at 100–125 ms. Taken together previous TMS and the present MEG findings suggest that processing fearful expressions is associated with rapid parietal and frontal activations and future work may try to explore neural interactions within such parieto-frontal network. A similar picture emerges from a recent report of rapid amygdala responses to threat / harm body postures^20^.

A related issue is whether this early dorsal route activation is associated with conscious perception of the stimuli. The occipital and parietal lobes of the human brain contain two major processing streams: the ventral one is involved more in processes related to object recognition, and the dorsal one more in spatial processing, attention, and online control of actions^21^. This two-stream view does not imply an absolute division, and processing of some object categories strongly involves both streams. For example tools trigger activity related to the object category in ventral areas, but also to action-observation-execution in dorsal areas^22^,^23^. The issue of the relation between stimulus awareness, dorsal stream activity and perception of emotional body images has not been addressed in the literature except for the hemianopic brain^24^,^25^. In line with the mainstream view on category specific processing of body stimuli, the ventral body selective area is most often considered as the gateway to subsequent processing of various body attributes such as perception emotion expression. Indeed, an ERP time course study^26^ reported the earliest body stimulus sensitivity in the time window corresponding to the N170 component and did not find a temporal modulation of the N170 by the fear expression. However, a later study^27^ used stimuli that were matched for the action displayed (opening a door in a neutral vs. in a fearful fashion) and found faster processing of fearful body expression as compared with neutral body expression for two early ERP components, i.e. the P1 component around 110 ms and the VPP component around 175 ms. Interestingly, two recent studies showed that the N170 is larger for fearful than for neutral or happy body postures^28^,^29^.

The matter of speed of processing and dorsal stream activation must be considered independently of that of stimulus awareness. Consistent with the early dorsal activation found in the present study, we recently observed that, unlike in ventral route structures, activity in the dorsal route structure IPS was not sensitive to the difference between consciously seen or unseen body images^30^.

A further issue is whether the observed results generalize to other whole body expressions besides the fear one used here. Based on previous fMRI findings^31^ we predict that anger expressions would yield the same results because, like fear expression, they represent a threat to the viewer. Further research is needed to clarify this conjecture.

In conclusion, the present findings provide the first empirical evidence for the hypothesis that, in addition to the subcortical tectopulvinar system^15^,^32^, the dorsal route structures have a functional role in the rapid detection of threatening stimuli^3^,^33^, and mediate a fast link between affective vision and action^4^ before detailed analysis in the ventral stream is completed.

## Methods

### Participants

Nine healthy right-handed individuals (mean age 28.0 years, range 22 to 37 years; four females) with normal or corrected to normal vision volunteered to take part in the experiment. All procedures were approved by the Massachusetts General Hospital Institutional Review Board, and informed written consent was obtained from each participant. The study was performed in accordance with the Declaration of Helsinki.

### Stimuli

**Figure 3:**
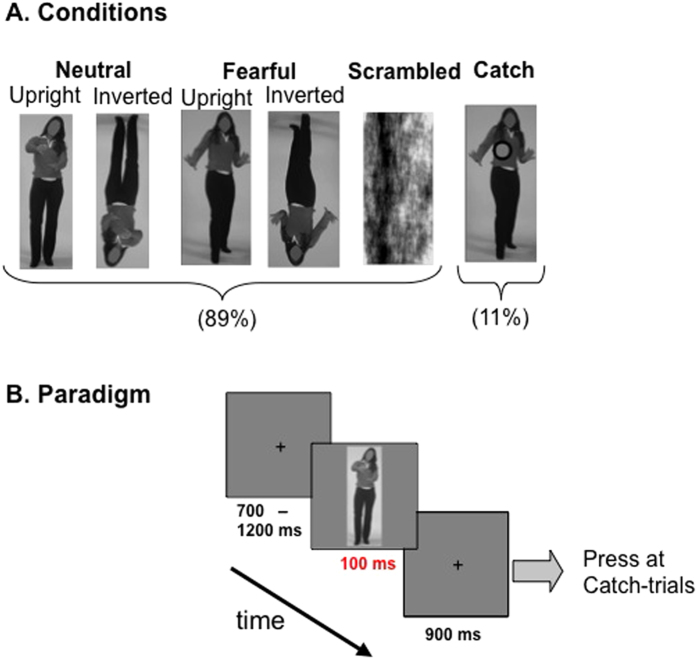
Experimental design. (**A**) Experimental conditions with examples of the visual stimuli. (**B**) Stimulus presentation paradigm. Subjects were instructed to make a button press at the appearance of a grey dot during Catch trials. Hence, the body stimulus and its emotional expression were task-irrelevant. Only trials without button press were analyzed.

Body stimuli were taken from our own validated dataset, previously used in behavioral^6^,^34^, EEG^26^ and fMRI studies^5^,^35^. They consisted of gray-scale images of whole bodies (4 males, 4 females) adopting a neutral or a fearful instrumental posture in which the faces were made invisible (for details see^35^). Stimuli were processed with photo-editing software in order to equalize contrast, brightness, and average luminance. To create control stimuli that contain the same spatial frequencies, luminance and contrast as their originals, all photographs were phase-scrambled using a two-dimensional Fast Fourier Transform. After randomizing the phases, scrambled images were constructed using the original amplitude spectrum. All images (photographs and scrambles) were pasted into a gray square (with an equal average gray value as the photographs), such that the final size of all stimuli was the same. Examples of the stimulus conditions can be found in Fig. 3A. Note that these images were previously used in an fMRI study^5^ and on that occasion a pilot experiment was run with a group of subjects (n = 10, six women, 24–33 years of age) to obtain data on evoked movement impression. The images were presented one by one in random order and subjects were instructed to rate the movement information on a five-point scale (from 1 for the weakest impression to 5 for the strongest). The mean ratings for the three categories were nearly identical (neutral, 3.5; fearful 3.5; and happy, 3.3). The possibility that the obtained differences in activation level were artifacts of differences in subjectively evoked movement can therefore safely be discarded.

### Experimental design

The experiment was run as a single session divided into two blocks with a short break (duration self-paced of ca. 30 sec-1 min) in between, during which participants were asked to remain seated with their heads still, but were allowed to blink or close their eyes according to their own convenience. There were five experimental stimulus conditions: Upright Neutral, Upright Fearful, Inverted Neutral and Inverted Fearful Bodies, and Scrambled Bodies (Fig. 3A). Each block consisted of 360 trials (320 experimental and 40 catch trials). Each block included one catch trial per stimulus variation (8 exemplars * 5 stimulus conditions) in addition to eight repetitions of every stimulus variation (ratio Catch-trials/Experimental-trials is 1:8), resulting in a total of 128 experimental trials for each stimulus condition over two blocks. To familiarize the subjects with the procedure and task demands the experiment was preceded by a short training session, which contained samples of all stimulus conditions.

The experiment was conducted in a magnetically shielded room (Imedco AG, Hägendorf, Switzerland). Subjects were comfortably seated with the head leaning against the back of the helmet of the MEG dewar. The visual stimuli were presented with a LP350 Digital Light Processor projector (InFocus, Wilsonville, OR) onto a back-projection screen placed 1.5 m in front of the subject. The size of the body images on the screen was 10 × 40 (width × height) cm, subtending a visual angle of 3.8° horizontally and 15.2° vertically. The trial designation is depicted in Fig. 3B. The stimuli were presented for 100-ms with an interstimulus interval that ranged between 1600–2100-ms. Participants were instructed to minimize eye blinks, head movements and all other movements. The participants’ task was to keep their eyes fixed on the cross and to press a button as accurately and fast as possible upon catch trials, i.e. the appearance of a gray dot superimposed onto the image of any of the five conditions. Participants changed the response hand between blocks; half of the subjects started responding with their left hand, while the other half started with their right hand. Hence, attention to the visual stimuli was maintained while the bodily expression was task irrelevant. The catch trials were discarded from analysis, hence signals were not contaminated with motor activity.

### MEG data acquisition

MEG data were acquired with a 306-channel Neuromag VectorView system (Elekta-Neuromag Oy, Helsinki, Finland), which combines the focal sensitivity of 204 first-order planar gradiometers with the widespread sensitivity of 102 magnetometers. Eye movements and blinks were monitored with vertical and horizontal electrooculogram (EOG). The location of the head with respect to the sensors was determined using four head-position indicator coils attached to the scalp. A head-based MEG coordinate frame was established by locating fiduciary landmarks (nasion and preauricular points) with a Fastrak 3D digitizer (Polhemus, Colchester, VT). The data were digitized at 600 samples/second with an anti-aliasing low-pass filter set at 200 Hz.

MEG signals were averaged across trials for each condition, time-locked to the onset of the stimulus. A 34-ms delay between the time the computer sent an image and the time it was projected onto the screen was measured with a photodiode and subsequently taken into account when reporting the timing of measured activity. A 200-ms pre-stimulus period served as baseline. Trials to which subjects made an incorrect response and those that contained eye blinks exceeding 150 μV in peak-to-peak EOG amplitude or other artifacts were discarded from the average. The evoked responses were low-pass filtered at 40 Hz.

### Structural magnetic resonance imaging (MRI)

MEG data were co-registered with structural high-resolution magnetic resonance images (MRI). A set of 3-D T1-weighted MR images using a 1.5 T system were acquired. The MRI and MEG coordinate systems were aligned by identifying the fiducial point locations in the MRIs. In addition several points were digitized from the head surface to allow confirmation and fine tuning of the initial alignment based on the fiducial landmarks.

The geometry of the cortical mantle was extracted from the MRI data of each individual subject using the Freesurfer software^36^,^37^. An inflated representation of the cortical surface was used for visualization to allow viewing the gyral pattern and the cortex embedded in fissures.

### MEG source estimation

The source current distribution was estimated in each individual participant at each cortical location using the minimum-norm estimate (MNE)^38^. The cortical surface was sampled with ca. 5000–7000 dipoles at the interface between gray and white matter provided by Freesurfer with an average 7-mm spacing between adjacent source locations. The forward solution for each of the three dipole components at each of these locations was computed for all 306 sensors using an anatomically realistic single-layer Boundary Element Model^39^. The inner skull boundary for this model was derived from each subject’s MRI. The strength of the fixed-location sources was estimated for each time-instant of the evoked response applying the linear inverse solution using a cortical loose orientation constraint^40^. The resulting current amplitudes were noise-normalized by dividing the magnitude of the estimated currents at each location by their respective standard deviations^7^. The latter was estimated with help of the spatial noise-covariance matrix, which was computed from the 200-ms pre-stimulus activity in the non-averaged data set with the same filter settings as for the evoked responses. This noise-normalization procedure reduces the location bias towards superficial currents, inherent in the minimum-norm solution, and equalizes the point-spread function across cortical locations^7^. The noise-normalized solution provides dynamical Statistical Parametric Maps (dSPM), which essentially indicate the signal-to-noise ratio of the current estimate at each cortical location as a function of time. Thus, dSPM movies of brain activity are useful for visualization of the data as they identify locations where the MNE amplitudes are above the noise level.

*Group movies* were created by morphing the source estimates for each individual subject to the cortex of one representative subject, according to the method of Fischl *et al*.^41^. Subsequently, the values were averaged across individuals at each source location. The dSPM values were used to identify spatiotemporal cortical patterns that show consistent responses across individuals.

#### Statistical Analysis

In order to make statistical inferences on the source level we tested the resulting dSPM values for significant differences between the fearful and neutral condition (i.e. fearful > neutral) across subjects (random effects). Nonparametric randomization tests based on spatiotemporal clustering^8^ were performed using the “FieldTrip” open-source toolbox^42^ and custom software. By clustering neighboring cortical locations and subsequent time points that show the same effect, this test deals with the multiple comparisons problem while taking into account the dependency of the data. As a first step, for each cortical point a paired-samples t-value was computed (testing the fear-neutral contrast >0). Second, all samples were selected for which this t-value exceeded an a priori threshold (uncorrected p < 0.05). Third, the selected samples were clustered on the basis of spatial and temporal adjacency (a sample was only included when there were at least three neighboring samples in space or time), and the sum of the t-values within a cluster was used as cluster-level statistic. The cluster with the maximum sum was used as test statistic. By randomizing the data across the two conditions and recalculating the test-statistic 1000 times, we obtained a reference distribution of maximum cluster t-values to evaluate the statistic of the actual data.

### MNE time courses

The time courses of the estimated MNE values for each dipole within the significant cluster were extracted and used for further analysis. One-tailed *t*-tests for paired samples (fear - neutral >0) were performed on the mean current strength across dipoles for the upright and inverted conditions at successive time points.

## Additional Information

**How to cite this article**: Meeren, H. K. M. *et al*. Early Preferential Responses to Fear Stimuli in Human Right Dorsal Visual Stream - A Meg Study. *Sci. Rep.* **6**, 24831; doi: 10.1038/srep24831 (2016).

## Figures and Tables

**Figure 1:**
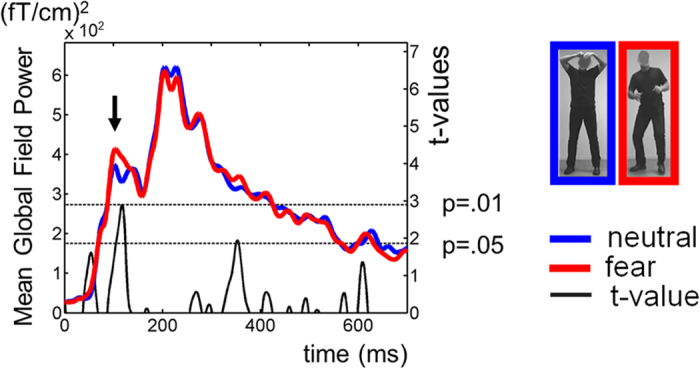
Mean global field power (MGFP) of the MEG signal evoked by photographs of upright neutral (blue) and fearful bodies (red). There is an increased response to fearful bodies around 100-ms after stimulus onset. Shown are the grand averages and corresponding t-values (black line, right vertical axis) for the contrast Upright Fear > Upright Neutral. The dotted black horizontal lines indicate t-levels corresponding to p-values of 0.05 and 0.01.

**Figure 2:**
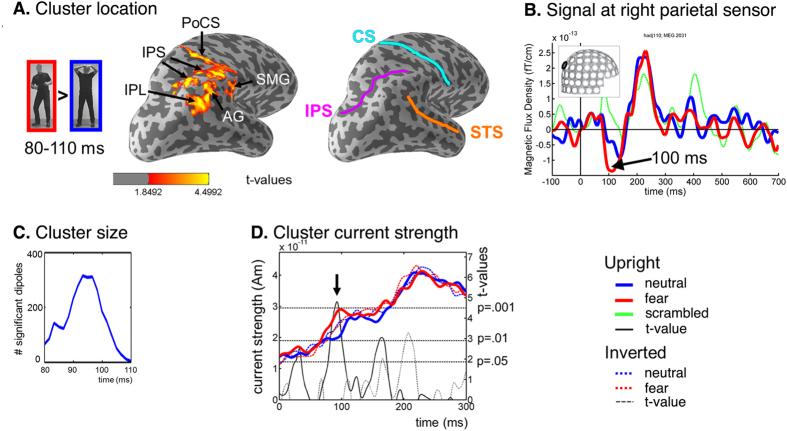
Right parietal cluster in response to upright fearful bodies in the 80–110-ms time window. (**A**) Cortical distribution of the spatiotemporal cluster that responded stronger to upright fearful than upright neutral bodies between 80–110 ms (*P* = 0.012) on the inflated cortical surface of the right hemisphere (lateral-occipital view), with main sulci indicated on the right (CS = central sulcus; STS = superior temporal sulcus; other abbreviations in text). (**B**) Signal at the right parietal sensor for each condition (blue: neutral; red: fear; green: scrambled stimuli) showing fear effect at the sensor level at ~90–100 ms. (**C**) Time course of cluster size in number of significant dipoles included in the cluster. (**D**) Time courses of average current strength across all cluster dipoles (left vertical axis) with corresponding t-values (right vertical axis) for the fear effect (i.e. black straight line for upright fear > neutral; black dotted line for inverted fear > neutral). There is a strong fear effect (*P* < 0.001) around 95-ms after stimulus onset, but only for the upright images, not for the inverted images. The dotted black horizontal lines indicate t-levels corresponding to p-values of 0.05, 0.01, and 0.001.
